# The prevalence and determinants of non-communicable diseases among Ghanaian adults: A survey at a secondary healthcare level

**DOI:** 10.1371/journal.pone.0281310

**Published:** 2023-02-08

**Authors:** Hosea Boakye, Albert Atabila, Thomas Hinneh, Martin Ackah, Folasade Ojo-Benys, Ajediran I. Bello

**Affiliations:** 1 Physiotherapy Department, LEKMA Hospital, Accra, Ghana; 2 Department of Biological, Environmental and Occupational Health, School of Public Health University of Ghana, Accra, Ghana; 3 School of Health Sciences, University of Dundee, Dundee, Scotland, United Kingdom; 4 Tain District Hospital, Ghana Health Service, Nsawkaw, Ghana; 5 Physiotherapy Department, Korle-Bu Teaching Hospital, Accra, Ghana; 6 Public Health Department, LEKMA Hospital, Accra, Ghana; 7 Department of Physiotherapy, School of Biomedical and Allied Health Sciences, College of Health Sciences, University of Ghana, Accra, Ghana; Federal Medical Centre Umuahia, NIGERIA

## Abstract

The current epidemiological transition of diseases in Ghana necessitates understanding their burden and the associated context-specific risk factors to inform disease prevention strategies. To determine the prevalence and determinants of selected Non-Communicable Diseases (NCDs) among patients seeking healthcare services in a secondary health facility in Ghana. A facility-based survey was conducted among adult patients 18 years and above between May and July 2021, using a multi-stage sampling approach. Data regarding the prevalence of NCDs, participants’ socio-demographics and lifestyle factors of NCDs were obtained using Modified STEPwise Approach to NCD Risk Factor Surveillance (STEPS). The Chi-square test and regression analysis were performed to identify the risk factors of NCDs at P < 0.05. The participants comprised 480 patients with a mean age of 37.7±16.5 years, and 57.7% (277/480) of them were females. The overall prevalence of the selected NCDs was 26.7% (CI = 0.23–0.31), of which hypertension (22.7%) was the most prevalent. More than half (54.2%) of the participants engaged in alcohol consumption and 54% were physically inactive. The odds of developing NCDs were higher in females (CI = 1.32–4.10, P = 0.004), older adults (CI = 4.11–20.68, P <0.001), overweight/obese adults (CI = 1.65–4.70, P < 0.001), family history (CI = 0.15–0.46, P<0.001), and alcohol consumption (CI = 0.12–0.40, P < 0.001). There was an overall high prevalence of NCDs, strongly influenced by the participants’ age, sex, BMI, alcohol consumption, and family history. These determinants should be highlighted as part of the campaign for preventive action plans.

## Introduction

Non-communicable diseases (NCDs) have become diseases of public health concern, significantly affecting populations and health systems. Previously, NCDs were thought to be diseases of affluence, probably due to low levels of awareness and control rates among the less advantaged groups [1]. Today, NCDs account for about 71% of deaths globally every year, and has been reported to be higher than infectious diseases [2]. Of 41 million people who died from NCD-associated death, more than 15 million of them are between the ages of 30 and 69 years, thus constituting the productive population [3].

Countries in sub-Saharan Africa are at the centre of the raging consequences of NCDs [4, 5]. Nearly 77% of all NCD-related deaths occur in Low- and Middle-Income Countries (LMICs) [3]. More significantly, about 85% of all premature deaths caused by NCDs, also occur in LMICs [6]. In addition, there is a considerable increase in Disability-Adjusted Life-Years (DALYs) from 90.6 million DALYs in 1990 to 151.3 million DALYs in 2017 as a results of NCDs [7]. Undeniably, the Africa Region is undergoing an epidemiological transition regarding the burden of diseases. The fragile nature of the health systems in Africa coupled with the scarcity of capital and human resources for healthcare delivery system, are the potential factors undermining the implementation of sustainable health interventions.

Cardiovascular diseases and diabetes contribute to the largest national NCD burden in Ghana [8]. Indeed, about 65% of deaths in Ghana are associated with NCDs [9]. In the last decade, the population dynamics of Ghana has changed significantly owing to the gains in health systems strengthening interventions. The rising burden of NCDs in the country is associated with increasing population, improvement in life expectancy and the rising NCD risk factors [10]. Non-communicable diseases are related in a common pathway with notable risk factors such as unhealthy diets, physical inactivity, tobacco use, and harmful alcohol use. The World Health Organization Global Health Risk Estimation Report identified the following as the leading risk factors of global mortality: obesity (5%), physical inactivity (6%), raised blood glucose (6%), and tobacco use (9%) with hypertension alone, accounting for about 13% [11].

Evidence on NCDs prevalence and the associated risk factors informs primary health care strengthening and orientation towards prevention and treatment strategies. The secondary health care facilities are commonly patronized by individuals presented with various NCDs in Ghana. While the primary health care system is still undergoing transformation, it may be worthwhile to determine the risk factors influencing the NCDs prevalence at the secondary health care level. Studies on NCDs prevalence and their determinants tend to focus largely on cardiovascular diseases and diabetes [12–14], without corresponding attention to other forms of NCDs. Besides, there is a wide variation in methodologies and settings regarding the previous studies on risk factors and prevalence of NCDs in Ghana.

The present study adopted a holistic approach to examine the prevalence of selected NCDs (hypertension, diabetes, heart diseases, dyslipidemia, stroke, chronic obstructive pulmonary disease and cancer) and risk factors (physical inactivity, harmful use of alcohol, smoking and sedentary lifestyle) among adults attending a district hospital in Ghana.

## Method

### Study design/setting

We conducted a prospective, cross-sectional and institution-based survey among adults accessing healthcare services at Ledzokuku-Krowor Municipal Assembly (LEKMA) Hospital in the Greater Accra Region of Ghana. Adult patients were recruited from the hospital’s OPD during the time of data collection. The LEKMA Hospital is a 140-bed capacity facility and offers general and specialised services. It records an average adult outpatient attendance of about 8000 of all ages in a month. About 6000 of such attendants are adults aged 18 years and above. The study was conducted at the outpatient department (OPD) of the District Hospital from 8^th^ May to 31^st^ July 2021. The 2020 review of total attendants with NCDs indicated upward trend of the OPD reported cases, which favours the choice of the hospital for this study.

### Participants’ recruitment and sampling strategies

The study included all persons from 18 to 64 years old who attend the OPD of LEKMA Hospital and gave their consent to freely participate. We excluded the following categories of persons from the study: Those who were wheelchair bound and could not participate in the recommended PA level, persons with cognitive or communication impairments, persons who were undergoing an exercise programme prescribed by a health professional for therapeutic benefits, as well as pregnant women.

**Fig 1:**
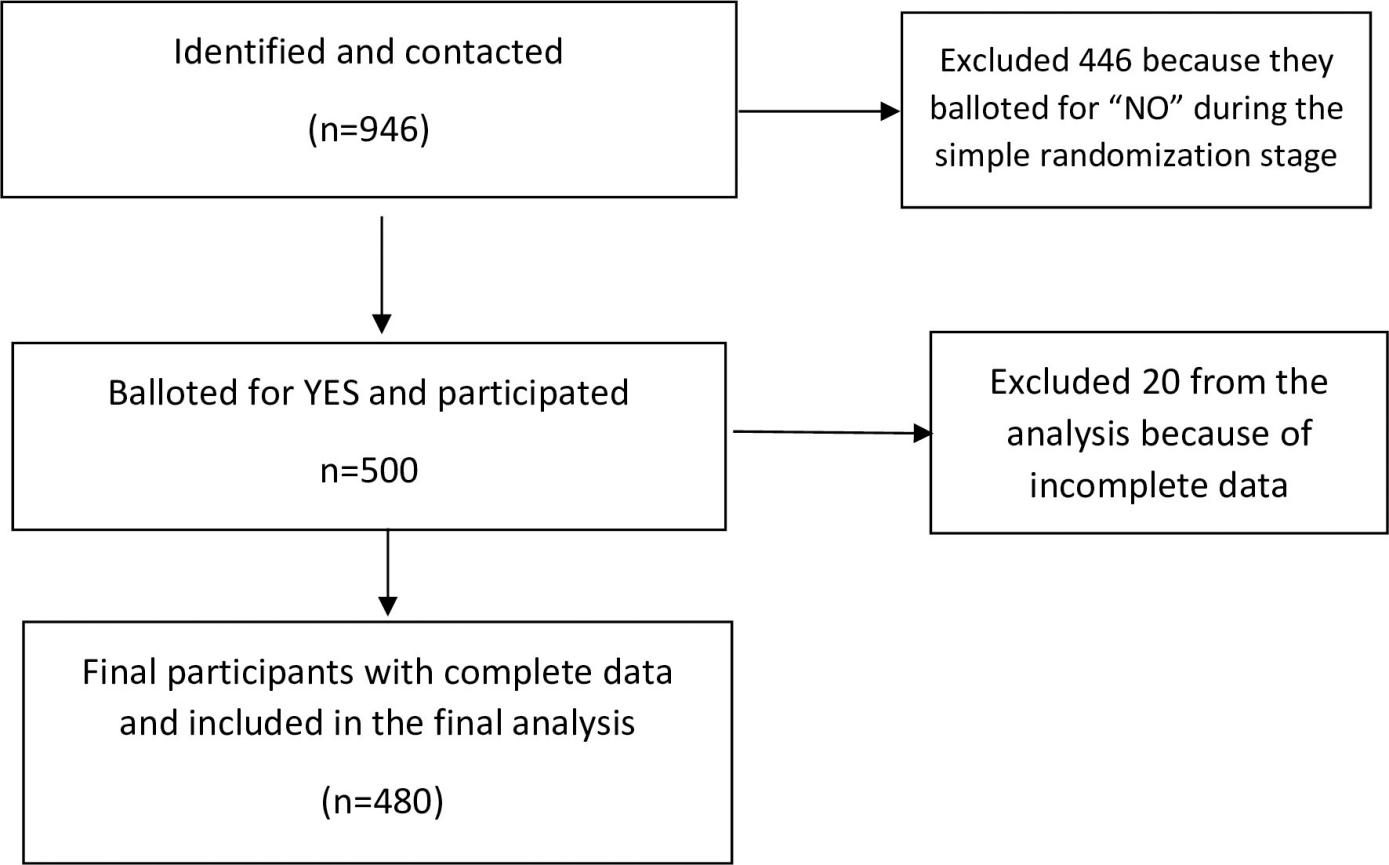
Flow chart for the recruitment of participants.

A multi-staged sampling method was adopted to recruit the participants. Using a stratified sampling technique, the participants were divided into three strata using age intervals (i.e., 18–34, 35–49, and 50–64). We further utilized a quota sampling strategy based on the 2021 outpatient attendance data available in the District Health Information Management Systems (DHIMS) in the ratio of 4.5:3.5:2, respectively. In the final stage of the recruitment, a simple random sampling technique was adopted to select eligible participants through balloting using wrapped pieces of paper with the inscription YES or NO from a container. The wrapped pieces of paper were kept in an opaque box and made available on each day of data collection. The participants’ recruitment process is available on the flow chart in Fig 1.

### Sample size estimation

Using the Taro Yamane method (1976) *n* = *N*/(1 + *N e*^2) where N is the total population, given as the average outpatient attendance for April, May and June (N = 6253), and e is the precision level (level of significance (0.05)). The authors estimated the minimum sample size of n = 376.

### Study measures

#### Outcome variable

The main outcome variable was the prevalence of NCDs among the participants reporting to LEKMA hospital. They responded to the question: *’Have you been previously diagnosed with any of the following conditions (hypertension*, *diabetes*, *dyslipidemia*, *heart attacks*, *stroke*, *cancers and Chronic Obstructive Pulmonary Disease’)*?. The binary responses were; Yes = 1 and No = 0.

#### Exposure variables

The exposure variables were categorized as; socio-demographic (age, sex, marital status, education, number of children, occupation, tribe, residence, body mass index (BMI), and monthly income) and lifestyle factors such as alcohol consumption, smoking status, physical activity level, and sedentary behavior.

### Data collection

#### Questionnaire

The study adopted and modified the STEPwise Approach to NCD Risk Factor Surveillance (STEPS) tool to explore variables relating to the risks of NCDs in Ghana [15] as well as the Global Physical Activity Questionnaire (GPAQ) to measure the physical activity level and sedentary behavior among the participants. The domains of the questionnaire were categorised into socio-demographic characteristics of participants, lifestyle factors, self-reported NCD status, and NCD risk factor assessment. The questionnaires have successfully been used in previous studies in Ghana with good internal and external validity [16, 17].

#### Procedure

This study was approved by the Ghana Health Service Ethical Review Committee on Research involving Human Subjects (GHS-ECR 026/08/21). Permission was sought from the Management and Head of the OPD of the LEKMA Hospital. Written informed consent was obtained from study participants before enrolment. The participants were made aware of the voluntariness of the study and they were made aware that they could withdraw at any time without any consequences. Three Research Assistants collected the data after two days of training prior to the exercise. The method of data collection was researcher-administered to elicit the required information from the eligible participants. All the participants were contacted after going through hospital registry while waiting for their turn to see the doctor. After explaining the rationale of the study, those who agreed to take part in the study balloted by picking wrapped pieces of paper in an opaque container. Those who picked ‘YES’ were given the informed-consent form to sign or thumb-print. The research assistants then administered the questionnaire which lasted for about 10–15 minutes. Covid-19 protocols were adhered to by wearing facemasks and social distancing throughout the data collections.

### Statistical analysis

All the data collected were entered directly into Microsoft Excel version 2016, cleaned, coded, and imported into Stata IC 16 for analysis. We descriptively presented the results using frequencies, percentages, mean and standard deviation (SD). Bivariate analysis was performed using Chi-square to test the association between NCDs, socio-demographic characteristics and risk factors. To compare the magnitude of the risk factors, the variables that were statistically significant in the above test were entered into the multivariable logistic regression to estimate the adjusted Odds Ratio (AOR). A significant level was set at P < 0.05 with corresponding 95% Confidence Interval (CI) for all the statistical tests.

## Results and discussion

### Demographics

Out of the 500 copies of the questionnaires administered, 480 copies were validly completed and were used for the final data analysis. Two hundred and three (42.3%) were males and 277 (57.7%) were females. Their ages ranged from 18 to 64 years with a mean age of 37.7±16.5 years. The highest proportion of the respondents (46.5%) were from the age group 18–34 years., and most of them were from the Ga-Adangbe (41.3%) and Akan (33.8%) tribes. Two hundred and twenty-nine (47.7%) were in marital union, and 94% of the respondents had formal or vocational education, with 30 (6%) having no formal education.

As regards the participants’ employment status, 406 (84.6%) were employed, while 74 (15.4%) were unemployed. The monthly income level of respondents was dichotomised into above and below 1000 Ghana Cedis. A greater number of the respondents fall below 1000 Ghana Cedis, (316; 65.8%) compared to 164 (34.2%) who earned above 1000 Ghana Cedis as shown in Table 1.

**Table 1:** Socio-demographic characteristics of participants (n = 480).

Socio-demographic characteristics		Total N (%)
Age	18–34	223 (46.5%)
	35–49	176 (36.7%)
	50–64	81 (16.8%)
Marital status	Not in union	251 (52.3%)
	In union	229 (47.7%)
Tribe	GA-Adangbe	198(41.3%)
	Akan	162(33.8%)
	Ewe	102(21.3%)
	Mole-Dagbani	18 (3.8%)
Education Status	No formal education	30 (6.3%)
	Primary	158 (32.9%)
	Secondary	153 (31.9%)
	Technical/vocation	56 (11.7%)
	Tertiary	83 (17.2%)
Occupation	Unemployment	74 (15.4%)
	Employed	406 (84.6%)
Monthly Income	Below 1000GHC	316 (65.8%)
	From 1000 and above	164 (34.2%)

#### Prevalence of NCD Risk factor

*Family history*. The prevalence of NCD risk factors is presented in Table 2. Overall, more than half (58%) of the respondents exhibited a family history of one or more NCDs. Out of this, 142 (50.7%) reported one, 98(35%) reported two, 22(7.9%) reported three, 11 (3.9%) reported four and 7(2.5%) reported five risk factors.

**Table 2:** Prevalence of NCDs and risk factors among adult population in LEKMAH by sex (n = 480).

NCD/RISK FACTOR	Response	Gender		Total N (%)	P-value
		Males No. (%)	Females No. (%)		
Smoking	Yes	33 (16.3%)	12(4.3%)	45 (9.4%)	**0.000**
	No	170(83.75)	265(95.7%)	435(90.6%)	
Alcohol Consumption	Yes	123(60.1%)	137(49.5%)	260 (54.2%)	0.160
	No	80(39.9%)	140(50.5%)	220 (45.8%)	
Physical Activity	Yes	127(57.2%)	76(29.5%)	222(46.25%)	**0.000**
	No	95(42.8%)	182(70.5%)	258 (53.75%)	
Sedentary lifestyle	Less than 5hrs	35(17.2%)	17(6.6%)	80 (16.7%)	**0.000**
	More than/equal 5hrs	168(83.8%)	260(93.4%)	400 (83.3%)	
Overweight/Obese	Yes	61(30.0%)	115(41.5%)	176 (36.7%)	**0.010**
	No	142(70.0%)	162(58.5%)	304 (63.3%)	
Family History	Yes	117(57.6%)	163(58.8%)	280 (58.3%)	0.791
	No	86(42.4%)	114(41.2%)	200 (41.7%)	
Hypertension	Yes	32 (15.8%)	77 (27.8%)	109 (22.7%)	**0.002**
	No	171(84.2%)	200 (72.2%)	371 (77.3%)	
Diabetes	Yes	6 (3.0%)	13 (4.7%)	19 (4.0%)	0.335
	No	197(97%)	264 (95.3%)	461 (96.0)	
Dyslipidemia	Yes	3 (1.5%)	12 (4.3%)	15 (3.1%)	0.076
	No	200 (98.5%)	265 (95.7%)	465 (96.9%)	
Heart disease	Yes	4(2.0%)	3 (1.1%)	7 (1.5%)	0.504
	No	199 (98.0%)	274 (98.9%)	473(98.5%)	
Stroke	Yes	6 (3.0%)	4 (1.4%)	10(2.1%)	0.252
	No	197(97.0%)	273 (98.6%)	470 (97.9%)	
COPD	Yes	0 (0%)	2 (0.7%)	0 (0%)	0.225
	No	203 (100%)	275 (99.3%)	480(100%)	

*Alcohol consumption*. Two hundred and sixty (54.2%) have ever taken alcohol in their lifetime Table 2. Almost 53% of the participants who had taken alcohol before were females.

*Smoking*. The prevalence of smoking was 9.4%, of which males constitute 73% of those smoking Table 2. There was a statistically significant association between the history of smoking and sex. However, smoking had no significant association with suffering from an NCD Table 4.

*Physical inactivity*. Similarly, almost 54% of the respondents were also physically inactive. More than 80% of the respondent engaged in sedentary lifestyles for more than five hours. Males are likely to be more physically active and engaged in less sedentary behaviour compared to females, which was statistically significant Table 2. It was observed that sex has significant association with both physical activity and showing sedentary behaviour.

*Obesity/Overweight*. The overall prevalence of overweight/obesity was 36.7% of the study participants Table 2. The mean and standard deviation of the BMI of the participants was 24.02 ±4.26.

#### Prevalence of selected non-communicable diseases

The overall prevalence of selected NCDs among the respondents was 26.7% (CI = 0.23–0.31). Out of the 128 (26.7%) who reported having been diagnosed with the selected NCDs, 99 (77.3%) reported one NCD, 25 (19.5%) reported two, while 2 (1.6%) each reported three and four NCDs. The prevalence of the selected NCDs are: hypertension, 109 (22.7% (CI = 0.19–0.27)); diabetes, 19 (4.0% (CI = 0.0.03–0.06)); dyslipidemia, 15(3.1% (CI = 0.02–0.06)); stroke 10 (2.1%); heart diseases, 7 (1.5%); COPDs, 2 (0.4%). No report of previously diagnosed cancer was found as shown in Table 3. But for COPD, There were statistically significant differences in the participants’ hypertension, diabetes, stroke and dyslipidemia status by age groups. The prevalence of hypertension, diabetes, and dyslipidemia increases with advanced age Table 3. Nearly two-thirds of the participants with history of hypertension and diabetes were females Table 2. There was an observed statistically significant difference between sex and having hypertension but not diabetes Table 2.

**Table 3:** Prevalence of selected NCDs.

Selected NCDs	Response	Age Range			Total No. (%)	P-value
		18–34	35–49	50–64		
Hypertension	Yes	15 (6.7%)	46(26.1%)	48 (59.3%)	109 (22.7%)	**0.000**
	No	208 (93.7%)	130 (73.9%)	33 (40.7%)	371 (77.3%)	
Diabetes	Yes	5 (2.2%)	5 (2.8%)	9 (11.1%)	19 (4.0%)	**0.001**
	No	218(97.8%)	171 (97.2%)	72 (88.9%)	461 (96.0)	
Dyslipidemia	Yes	3 (1.3%)	5 (2.8%)	7 (8.6%)	15 (3.1%)	**0.005**
	No	220 (98.7%)	171 (97.2%)	74 (91.4%)	465 (96.9%)	
Heart Disease	Yes	2 (0.9%)	4 (2.3%)	2(2.4%)	7 (1.5%)	0.180
	No	221(99.1%)	172 (97.7%)	80 (97.6%)	473(98.5%)	
Stroke	Yes	0 (0%)	3 (1.7%)	7 (8.6%)	10(2.1%)	**0.000**
	No	223(100%)	173 (98.3%)	74 (91.4%)	470 (97.9%)	
Cancer	Yes	0 (0%)	0 (0%)	0 (0%)	0(0%)	**0.000**
	No	223 (100%)	176 (100%)	81 (100%)	480(100%)	
COPD	Yes	1 (0.4%)	1 (0.6%)	0 (0%)	2 (0.4%)	0.311
	No	223 (99.6%)	175 (99.4%)	80 (100%)	478 (99.6%)	

#### Determinants of NCD status

All the significant variables from the Chi-square analysis (Table 4) were selected for multivariable logistic regression analysis to estimate the AOR. The results indicate that the odds of females getting NCDs is 2.32 times that of males (AOR = 2.32, CI = 1.32–4.1, P<0.004). People aged 35 to 49 years are 3.03 times more likely to suffer NCDs than those aged 18 to 34 years (AOR = 3.03, CI = 1.61–5.69, P<0.001) and those aged between 50–64 years are 9.22 times more likely to suffer from NCDs compared to those aged 18 to 34 years (AOR = 9.22, CI = 4.11–20.68, P<0.001). People without family history of NCDs were 30% less likely to suffer from NCDs than those with family history of the diseases (AOR = 0.30, CI = 0.15–0.46, P<0.001). Obese or overweight people are 2.78 times more likely of developing NCDs than those with healthy weight (AOR = 2.78, CI = 1.65–4.70, P<0.001). People who have never consumed alcohol in their life time are 22% less likely of developing NCDs than their counterparts who have ever consumed alcohol (AOR = 0.22, CI = 0.12–0.40, P<0.001) Table 5.

**Table 4:** Association of NCD risk factors by NCD status.

Socio-demographic factors	Have you been diagnosed of NCDs			
	Yes	No	Chi-square	p-value
**Gender**				
Male	39	64	10.00	0.002*
Female	89	188		
**Age/years**				
18–34	23	199		
35–49	53	123	89.48	0.000*
50–64	52	29		
**Overweight/obese**				
**Yes**	71	105		
**No**	57	247	26.5715	0.000*
**Having a child**				
Yes	105	176	39.68	0.000*
No	23	176		
**Marital status**				
In union	79	49	13.73	
Not in union	150	202		0.000*
**Educational status**				
No formal Education	12	18		0.000*
Primary	64	94		
Secondary	28	125	30.23	
Technical/ Vocational	11	45		
Tertiary	13	70		
**Occupation**				
Employed	93	259	0.10	0.754
Unemployed	32	93		
**Tribe**				
Ga-Adangbe	58	140		
Akan	39	123	3.64	0.302
Ewe	29	73		
Mole-dagbani	2	16		
Monthly income				
Below Minimum	85	43	0.02	0.873
Above Minimum	42	121		
**Alcohol consumption**				
Yes	90	170	18.33	0.000*
No	38	182		
**Smoking**				
Yes	13	32	0.13	0.723
No	115	320		
**Family history of NCDs**				
Yes	101	179	30.39	0.000*
No	27	173		
**Participation in physical activity**				
Yes	42	180	12.68	0.000*
No	86	172		
**Sedentary lifestyle**			1.13	0.042*
**Less than 5hrs**	14	66		
**More than/equal to 5hrs**	114	286		

**Table 5:** Multivariate logistic regression to determine predictors of NCD risk factors (n = 480).

	Adjusted OR	95% CI	p-value
**Gender**			
Male	Ref		
Female	2.32	1.32–4.1	0.004*
**Age/years**			
18–34	Ref		
35–49	3.03	1.61–5.69	0.001*
50–64	9.22	4.11–20.68	0.000*
**Marital status**			
In union	Ref		
Not in union	0.75	0.43–1.29	0.306
**Do you have a child**			
Yes	Ref		
No	0.81	0.42–1.56	0.529
**Education**			
No formal education	Ref		
Primary	1.25	0.46–3.4	0.668
Secondary	0.60	0.21–1.69	0.332
Technical/vocational	0.60	0.18–2.03	0.414
Tertiary	0.77	0.24–2.54	0.676
**Occupation**			
Unemployed	Ref		
Employed	0.92	0.46–1.82	0.813
**Overweight/Obese**			
No	Ref		
Yes	2.78	1.65–4.70	0.000*
**Consumed alcohol before**			
Yes	Ref.		
No	0.22	0.12–0.40	0.000*
**Family history**			
Yes	Ref.	0.15–0.46	0.000*
No	0.30		
**Participation in PA**			
Yes	Ref.		
No	1.33	0.73–2.46	0.350
**Sedentary Behaviour**			
≤5hours	Ref		
>5hours	1.20	0.51–2.84	0.673

## Discussion

The overall prevalence of NCDs in this study was 26.7%. In the last decade, the burden of NCDs in Ghanaian society has increased significantly. The brevity of the present study is owed to the number of diseases investigated and the highlights of the burden of NCDs in a typical Ghanaian health care facility. Hypertension prevalence alone accounts for 22.7%. This rate falls within the interval of hypertension prevalence rate reported by several cross-sectional studies and systematic reviews conducted in Africa and Ghana [18–20]. A systematic review and meta-analysis that included 13 articles in sub-Africa found the prevalence of hypertension between 15% and 70% [18]. Specifically in Ghana, a systematic review and meta-analysis found a pooled prevalence of 30.1% with a range of 4.5% to 54.3% among populations living in both urban and rural settings [19]. Similarly, our study found the prevalence of stroke to be 2.1%. A previous study that assessed the prevalence and correlates of stroke among older adult Ghanaians reported the prevalence of stroke to be 2.6% [20]. Given the similarity between the ages of our participants and previous studies, it is quite apparent that the rate of stroke among young adults in Ghana is rising steadily. Therefore, pragmatic measures to curtail the upsurge in the prevalence among adults, especially in the working population is urgently needed.

### Prevalence of selected risk factors

Several studies have linked the development of NCDs to several factors. The factors include age, sex, income levels, educational status, alcohol use, smoking, air quality, access to quality healthcare, diet and physical activity [21–24]. Baird & Cooper [25], grouped these factors into genetic, environmental, behavioural, psychosocial, preconception and pregnancy.

The prevalence of lifetime alcohol consumption in this study was 54.2%. The prevalence of alcohol consumption seems to be generally high in Ghana. A study that assessed the prevalence and determinants of alcohol consumption among the youth in the Volta Region of Ghana reported a lifetime prevalence of 43.4% [26].

The high prevalence of alcohol consumption among Ghanaians can be ascribed to advertisement, peer influence and curiosity due to its supposed benefits such as sexual enhancement, appetiser, and relaxation [26]. In a contemporary Ghanaian society, males are believed to consume more alcohol than females, which could be attributed to societal norms where male drinking is considered normal and vice versa for the female counterpart [27, 28]. Moreover, some studies conducted in South Africa, India and Nepal corroborate this assertion [27]. Interestingly, unlike the findings in the above previous studies, 53% of people with a history of alcohol consumption were females in our study. With the rising spate of physical inactivity among women and postmenopausal hormonal changes, the risk of developing NCDs among women could increase. Putatively, the physiology of alcohol metabolism are different between men and women [28]. Women are known to have less total body water than their conuterparts who are men with the same body weight. Thus, consumption of the same amount of alcohol, would lead to higher blood alcohol concentration in women, thereby potentially putting women at high risk from alcohol-related NCDs [29]. In other words, women are easily intoxicated with small volumes of alcohol. Conversely, women eliminate alcohol faster than men which also confer an advantage of less risk of alcohol-related NCDs such as stroke, cirrhosis or some cancer among women. Thus, while there was high risk of harm from alcohol use, its remote effects regarding other NCDs were less likely to be observed among women as much as it would in men [30].

In the same vein, the prevalence of physical activity recorded in this study was 46.3%. There is a global decline in physical activity owing to urbanisation and technological advancements. Recently, this has been compounded by the COVID-19 pandemic, where measures to curtail its spread favours physical inactivity [31]. Previous studies by Oyeyemi et al., [32] and Afrifa-Anane et al., [33] recorded a higher prevalence of physical inactivity than the present study. The decline in physical activity in our study could be related to the impact of the COVID-19 pandemic, as reported in another similar study by Castañeda-Babarro et al., [31]. In our study, it is worth noting that sex plays a key role in physical activity participation with males being more physically active (57.2%) compared to (42.8%) females. This trend is consistent with the findings of the previous studies [32, 33].

In another dimension, the prevalence of overweight/obesity among the respondents was 36.7%. According to a WHO global report on obesity, the prevalence of overweight/obesity has doubled since 1980 [34]. It is estimated that 2.5 billion of the world’s adult population are overweight or obese [34]. A systematic review on obesity in Africa showed a higher prevalence of overweight/obesity among females than males [35]. Specifically in Ghana, Ofori-Asenso and colleagues in a systematic review and meta-analysis, demonstrated a rising prevalence of overweight/obesity among Ghanaians [36]. The prevalence rate of 43% reported among females in this previous study is lower than the 65.3% documented in our study.

### Determinants of NCDs

This study reveals significant associations between NCDs and sex, age, BMI, marital status, educational status, occupation, alcohol, family history, sedentary behaviour and physical activity.

Regarding sex, males are generally more involved in unhealthy lifestyles such as smoking and drinking alcohol than their female counterparts, while more females are physically inactive than males [37]. A comparative study conducted in China found women at a higher risk of developing hypertension, diabetes and heart diseases than their male counterparts. Conversely, men were at higher risk of developing stroke and chronic heart diseases than women [37]. The observation in this study agrees with that of Opoku et al., [38], whose findings also showed the life risk of hypertension for men at early stages as opposed to women due to the physiological protection women experience during the pre-menopausal stages. In this study, women have 2.5 times the risk of developing an NCD compared to men.

As reported by other studies, age emerged as a significant determinant of NCDs studied. The risk of developing an NCD increases steadily as one ages which is consistent with what other studies have reported recently in Ghana [39, 40]. Moreover, being overweight/obese increases the chances of developing NCDs by 2.78. Ofori-Asenso et al., [35], also found a strong tie between overweight/obesity and diabetes. Overweight/obesity is likely to rise in Ghana in the next decade due to the rising spate of urbanisation and the digital world where engagement in physical activities is drastically reducing. Furthermore, not having a family history of NCDs reduced the risk of developing an NCD to almost 27%. Similarly, the odds of developing NCDs in adults who have never consumed alcohol before is 0.22 times that of adults who have ever consumed alcohol. These findings align with the previous studies on this subject [10, 25]. According to the WHO reports on NCDs, effective control of NCDs demands focus on modifying the risk factors associated with the diseases. Moderate solutions are available for all stakeholders including the Governments, to reduce the common modifiable risk factors. Adequate monitoring of progress and trends of NCDs and their risk is important for formulating policy and priorities. To ameliorate the burden of NCDs on individuals and society, a holistic approach is demanded of all sectors. Thus, the interplay of various indicators such as health, finance, transport, education, agriculture, planning, and other factors are important to reduce the risks associated with NCDs, and to promote interventions to prevent and control them.

### Strengths and limitations

This study has significant strengths which are worth mentioning. It is one of the few studies to examine the burden of key NCDs at secondary healthcare levels in Ghana. This is important to inform the arrangement of health services for this special group of patients, given the rising burden of NCDs and associated complications. Again, the participants included in this study were from the various cultural, socioeconomic, urban and rural divides. This has positive implications for the diversity of the data collected and the generalizability of study findings.

Nonetheless, the study has limitations. We relied on previously diagnosed self-reported NCDs which might introduce a recall bias.

## Conclusion

Our study provided evidence of high prevalence of NCDs and their determinants among participants who access healthcare at LEKMA hospital at the time of this survey. Age, sex, BMI, alcohol consumption and family history, which are key determinants of the selected NCDs, heighten the need to strengthen health systems and health promotion activities to improve surveillance, treatment and control activities. This is particularly essential for primary healthcare facilities, which are generally accessible to many Ghanaian populations. Moreover, given the rising risk factors for NCDs, further research is required to investigate how to improve behavioural adherence to lessen the burden of NCDs in Ghana.

## Supporting information

S1 Data (XLSX)
